# A multi-dimensional pyramid strategy for limited sample classification of hyperspectral cropland imagery

**DOI:** 10.3389/fpls.2026.1773924

**Published:** 2026-03-04

**Authors:** Mingchao Yang

**Affiliations:** Comprehensive Geophysical Survey Team, Zhejiang Coal Geology Bureau, Hangzhou, China

**Keywords:** crop classification, feature fusion, feature pyramid, hyperspectral remote sensing, limited sample learning

## Abstract

Hyperspectral crop classification is often challenged by substantial intra-class spectral variability, high inter-class similarity, and the scarcity of high-quality labeled samples. These issues frequently lead to insufficient feature fusion or excessive computational complexity in conventional classification methods. To address these problems, this study proposes MDPC-Net, a limited sample hyperspectral crop classification method that couples a multi-dimensional pyramid with a Transformer architecture. The model extracts crop features from spectral, spatial, and joint spectral–spatial dimensions to capture fine-grained characteristics. A feature reorganization strategy is further incorporated to effectively reduce dimensional redundancy, while the Transformer modules enhance global dependency modeling, thereby improving the discrimination of crop features in complex environments. Comparative experiments with six classical models on three datasets—Matiwan Village, WHU-HongHu, and WHU-LongKou—demonstrate that MDPC-Net achieves competitive accuracy with substantially lower computational complexity, effectively balancing the trade-off between classification performance and efficiency. The proposed approach provides a promising solution for fine-grained hyperspectral crop classification under limited sample conditions.

## 1 Introduction

Precise crop classification forms the foundation for agricultural production management (Tang et al., 2024), policy formulation, and food security, providing essential information for decision support in agricultural systems (Guerri et al., 2024). Hyperspectral imagery (HSI), with its exceptionally high spectral resolution, captures subtle reflectance variations across continuous wavelength ranges, and has therefore been widely employed in hyperspectral image–based crop classification (HSICC) (Khan et al., 2024; Ullah et al., 2025).

However, crop classification remains challenging due to the intrinsic variability of agricultural ecosystems (Aasen et al., 2018). Growth-stage differences and stress conditions within the same crop species often lead to the “same object, different spectra” phenomenon, whereas spectral similarity among different crops in certain wavelength regions results in the “different objects, same spectrum” problem (Gallo et al., 2023). Both issues substantially reduce inter-class separability. Meanwhile, acquiring large quantities of high-quality, pixel-level annotated samples in farmland environments is costly and labor-intensive, making data scarcity a persistent bottleneck for supervised HSICC and motivating increasing interest in limited sample learning paradigms (Gao et al., 2017). Traditional machine learning approaches rely heavily on handcrafted spectral indices and texture descriptors for feature extraction and dimensionality reduction (Ali et al., 2023), followed by statistical or shallow learning algorithms such as support vector machines (SVM), random forests (RF), k-nearest neighbors (KNN), linear discriminant analysis (LDA), and naïve Bayes (NB) for classification. These methods depend strongly on expert experience in feature engineering and are prone to the “curse of dimensionality” when dealing with high-dimensional spectral data (Hamidi et al., 2021). Moreover, they typically treat spectral and spatial information independently and lack end-to-end joint learning capabilities (Konduri et al., 2020; Khan et al., 2023). As a result, they fail to model deeper spectral–spatial relationships, making it difficult to distinguish spectrally similar crop types in complex agricultural environments and limiting their ability to achieve fine-grained classification in real-world farmland scenes.

Deep learning methods have been increasingly applied to HSICC tasks in recent years and have achieved remarkable progress (Chandrasekharan et al., 2016). By constructing multi-layer nonlinear mappings, deep learning establishes an end-to-end learning mechanism capable of automatically capturing intricate spectral features and spatial contextual dependencies in hyperspectral data (Agilandeeswari et al., 2022). Convolutional neural networks (CNNs) are widely used to extract spectral–spatial features of crops. One-dimensional CNNs (1D-CNNs) focus primarily on spectral sequence modeling while neglecting spatial neighborhood information (Hu et al., 2015), limiting their ability to differentiate spectrally similar crops in farmland scenes with blurred boundaries or mixed pixels. Two-dimensional CNNs (2D-CNNs) can extract spatial textures but are insufficient in modeling complex inter-band spectral dependencies, which constrains their ability to capture subtle spectral differences among crop varieties (Nazeri et al., 2019). Three-dimensional CNNs (3D-CNNs) jointly extract spectral–spatial features but suffer from high computational complexity and require large amounts of high-quality annotated samples—conditions often unmet in real agricultural environments—thereby restricting their practical applicability (Fernandes et al., 2019). Moreover, CNN-based methods inherently emphasize local neighborhood information and often fail to capture global dependencies, making their predictions susceptible to salt-and-pepper noise.

To alleviate the dependence on large-scale labeled data, limited sample learning has recently emerged as an effective paradigm for remote sensing image classification limited sample limited sample approaches can be broadly categorized into three groups. Metric-based methods learn embedding spaces in which samples are classified based on similarity measures, enabling knowledge transfer from base classes to novel classes with limited samples. Meta-learning-based approaches aim to acquire task-agnostic initialization or learning strategies that can rapidly adapt to new classes under limited sample settings. More recently, transformer-inspired limited sample frameworks have been explored to leverage global contextual modeling and attention mechanisms for improved feature generalization. Despite their success in natural image domains, these methods face notable challenges when applied to hyperspectral data, including high spectral dimensionality, complex spectral–spatial coupling, and severe intra-class variability, which often lead to degraded generalization performance under extremely limited training samples.

To overcome these limitations and enhance global feature modeling in HSICC, researchers have explored improved CNN variants and Transformer-based architectures. (Roy et al. (2020) proposed HybridSN (Roy et al., 2020), a hybrid spectral CNN that integrates 3D-CNN and 2D-CNN layers to improve spectral–spatial feature representation while controlling model complexity. SqueezeNet (Iandola et al., 2016) employs pointwise convolutions to reduce feature dimensionality, followed by multi-scale convolutions, significantly compressing parameter counts. MobileNets (Howard et al., 2017) utilize depthwise separable convolutions to build lightweight neural networks by applying a single filter to each input channel, greatly reducing computational costs. Although these models achieve lower computational complexity, their classification accuracy remains limited. To enhance global context modeling, Wang et al. (2023) introduced ESSAN (Wang et al., 2023), which incorporates dilated convolutions and Transformer modules to improve large-scale feature perception and contextual representation (Tu et al., 2022). further introduced a pixel aggregation strategy that groups homogeneous regions and integrates them into a hierarchical Transformer framework, enabling adaptive multi-scale feature construction and more effective modeling of local spatial semantics (Michelon et al., 2023; Liang et al., 2024). These developments improve the model’s ability to perceive field shapes, crop distribution patterns, and critical spectral bands.

Despite recent advances, existing HSICC methods still struggle to simultaneously ensure low computational complexity and high classification accuracy. The growing demand for efficient and precise crop mapping in precision agriculture poses new challenges for model design and feature organization. SANet (Zhang et al., 2024) integrates spectral and contextual information while emphasizing intra-spectral autocorrelation. By combining spatial–spectral non-local blocks with multi-scale spectral self-attention (SSA), SANet allocates more attention resources to spatial and spectral dimensions and models inherent spectral–spatial correlations, thereby strengthening the representation of contextual structures and key spectral dimensions (Al Duhayyim et al., 2023) (Chen et al., 2024). introduced FrFSSPN, a frequency–spectral–spatial prototype network based on fractional Fourier transform, which integrates frequency-domain information with spectral–spatial representations to enlarge inter-class separability while preserving intra-class consistency. CMTNet (Guo et al., 2025) further enhances model robustness under limited sample conditions by incorporating a spectral–spatial feature extraction module for shallow features and enforcing cross-level constraints to improve classification stability (Lu et al., 2024; Guo et al., 2025).

Although these methods partially address data scarcity and global dependency modeling, most existing limited sample HSICC frameworks still rely on fragmented feature extraction pipelines or incur substantial computational overhead when integrating multi-dimensional information. In particular, the extremely high spectral resolution of hyperspectral imagery poses challenges for attention-based mechanisms, which may overemphasize long-range dependencies while neglecting local spectral–spatial structures. These limitations highlight the need for a unified and efficient architecture that can effectively balance representation capability and computational efficiency under limited sample settings.

Although these methods utilize CNN variants and Transformers to capture global dependencies and consider crop-specific growth variability, significant challenges remain for multi-channel hyperspectral tasks. Current HSICC models still suffer from fragmented feature extraction pipelines, making it difficult to effectively integrate features across multiple dimensions and thereby limiting their ability to capture complex spectral–spatial characteristics of crops. Moreover, as network depth increases, feature fusion modules often lead to substantial computational overhead, which restricts their adaptability to practical applications. Due to the extremely high spectral resolution of HSI, the multi-head self-attention (MHSA) mechanism may focus excessively on long-range global dependencies while overlooking local spatial structures. These limitations highlight the need for a unified architecture capable of balancing representation ability and computational efficiency.

To address these challenges, this study proposes a novel HSICC framework named MDPC-Net (Multi-Dimensional Pyramid Coupling Transformer Network), which integrates a multi-dimensional progressive feature extractor, a linear-projection pyramid fusion module, and Transformer-based global semantic modeling. First, three parallel progressive dilated convolution branches—1D, 2D, and 3D—are designed to extract spectral, spatial, and joint spectral–spatial features from different data dimensions. Second, the linear-projection feature pyramid reorganizes and fuses multi-dimensional features while reducing computational cost. Finally, Transformer modules leverage multi-head self-attention and multilayer perception mechanisms to capture global semantic dependencies across spectral–spatial domains.

The main contributions of this work are summarized as follows:

1. A multi-dimensional feature extraction backbone based on progressive dilated convolutions is proposed. Three parallel branches (1D, 2D, and 3D) capture hyperspectral features from different dimensions, while progressive dilation enables multi-level receptive field aggregation.
2. A linear-projection feature pyramid fusion module is designed. This module employs multi-scale depthwise separable convolutions to extract spatial features at different scales and reorganizes these features into a unified representation, enhancing multi-scale perception.
3. A unified architecture combining the multi-dimensional feature backbone, linear-projection pyramid, and Transformer is developed. This design effectively models global spectral–spatial dependencies and strengthens the discrimination of crops with similar spectral signatures. Experiments on three datasets demonstrate that MDPC-Net achieves high classification accuracy while maintaining low computational complexity.

## 2 Methods

### 2.1 Overall architecture of MDPC-net

**Figure 1:**
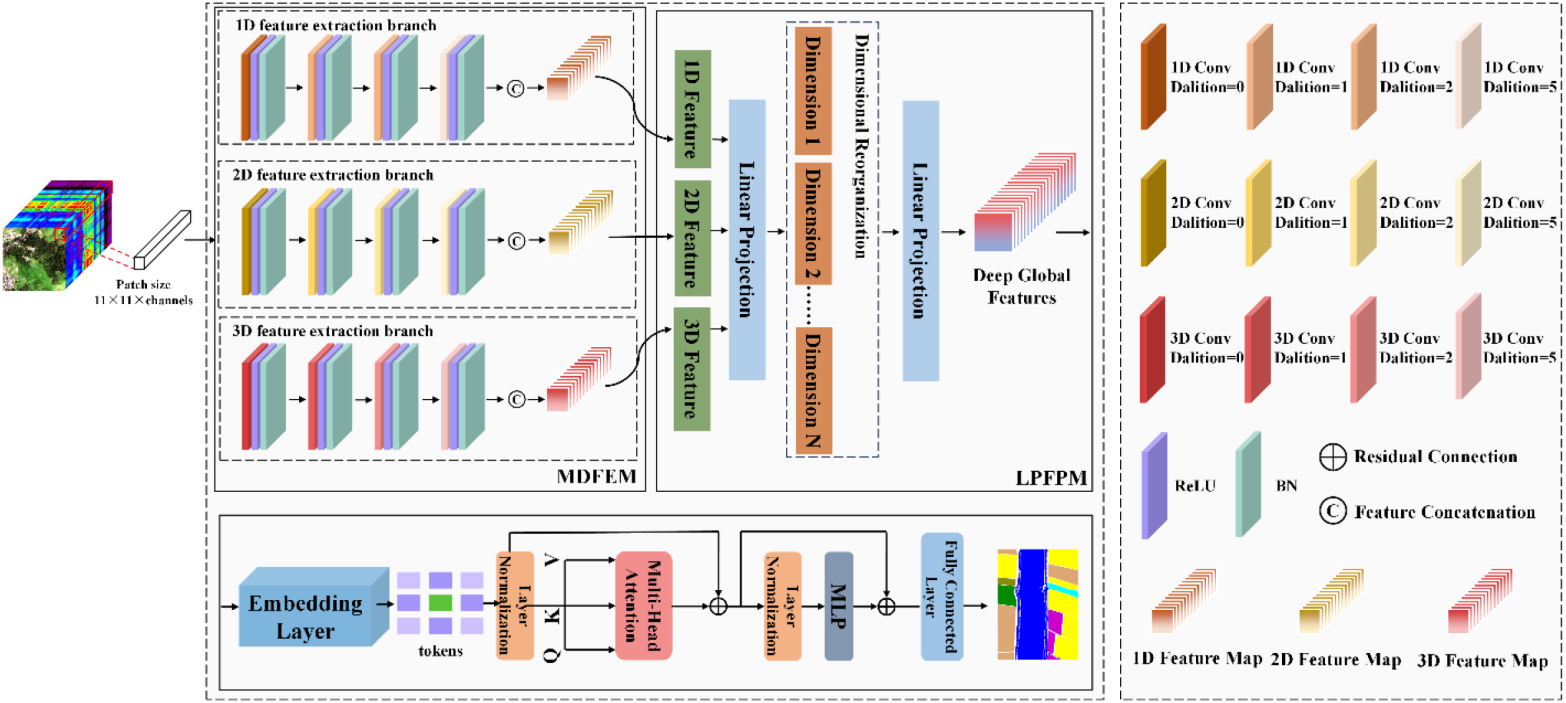
The overall network architecture of MDPC-Net.

The overall architecture of MDPC-Net is illustrated in **Figure 1**. The model consists of three components: A multi-dimensional feature extraction backbone based on progressive dilated convolutions, a linear-projection feature pyramid for multi-branch feature fusion, and an embedding and Transformer-based feature integration module for final representation learning and classification.

First, for each pixel in the original HSI, an image patch of size 11×11 is extracted and used as the basic processing unit. The patch is simultaneously fed into three parallel branches—1D, 2D, and 3D—to extract features from different data dimensions. All three branches employ progressive dilated convolutions, where zeros are inserted between convolutional kernel elements to gradually enlarge the receptive field without increasing the number of parameters or computational cost. This design enables the model to capture fine-grained local details while progressively learning broader global dependencies.

The feature maps obtained from the three branches are then forwarded to the linear-projection feature pyramid module. Through a linear mapping, features from all branches are projected into a unified dimensional space. The multi-dimensional features are then partitioned along the channel dimension into several groups for structural reorganization. A subsequent linear projection aggregates these reorganized groups to form deep, globally enriched representations.

Finally, the resulting global features are transformed into token sequences via an embedding layer and fed into the Transformer module. The Transformer captures long-range dependencies and facilitates comprehensive feature interactions across different scales, further enhancing the model’s ability to interpret complex spectral–spatial characteristics of crops in hyperspectral imagery.

### 2.2 Multi-dimensional feature extraction module

Conventional convolution is a fundamental operation in convolutional neural networks, where a kernel slides over the input with a predefined stride, and each kernel element is multiplied with the corresponding input element and summed to produce the output. The receptive field of standard convolution is determined by the kernel size. When larger-scale contextual information is required, the kernel size must be increased, which inevitably leads to a substantial growth in the number of parameters and computational cost. Furthermore, standard convolution extracts features at a single scale, making it insufficient for inputs containing multi-scale structures, as it may fail to capture comprehensive feature information.

Dilated convolution extends standard convolution by inserting zeros between kernel elements, where the dilation rate controls the spacing between sampled positions. This mechanism enlarges the receptive field without increasing the kernel size or parameter count, enabling the model to capture broader contextual information. However, using a fixed dilation rate restricts the receptive field to a single scale, potentially missing information at other scales and leading to the so-called “gridding effect,” which degrades feature extraction performance.

Equations 1, 2 present the effective kernel size and receptive field (RF) size of dilated convolution, respectively:

(1) $$\begin{array}{c} L=l+(l-1)(d-1) \end{array}$$

(2) $$\begin{array}{c} {\text{R}}_{m+1}={\text{R}}_{m}+(L_{m+1}-1)\times \prod_{i=1}^{m}{\text{S}}_{i} \end{array}$$

where L denotes the effective kernel size, l represents the size of the original convolution kernel, and d is the dilation rate. $R_{m+1}$ denotes the receptive field size of the (m+1)-th dilated convolution layer, and $S_{i}$ represents the stride of the i-th layer.

The Multi-Dimensional Feature Extraction Module (MDFEM) adopts a multi-scale representation strategy based on Progressive Dilated Convolution (PDC). The core idea of PDC is to construct hierarchical receptive fields through an increasing sequence of dilation rates. In our design, a dilation rate sequence of d=[0,1,2,5] is employed, which mathematically forms a receptive field pyramid. To further enhance the model’s capability in capturing diverse characteristics of hyperspectral data, three parallel branches—1D, 2D, and 3D—are incorporated to extract features from different dimensional perspectives.

The 1D branch applies one-dimensional convolution to focus on spectral-domain features, enabling the extraction of subtle variations along the spectral signatures. The 2D branch employs two-dimensional convolution to learn spatial information, such as structural patterns and textural details within hyperspectral imagery. The 3D branch uses three-dimensional convolution to jointly model the spectral and spatial dimensions, thereby capturing integrated spectral–spatial representations. The outputs from the three branches are flattened along the spatial dimension and concatenated to form a unified feature tensor. The fused feature representations of each branch are formulated in Equations 3–5, respectively.

(3) $$\begin{array}{c} X_{1D}=Concat(G_{0},G_{1},G_{2},G_{5}) \end{array}$$

(4) $$\begin{array}{c} X_{2D}=Concat(F_{0},F_{1},F_{2},F_{5}) \end{array}$$

(5) $$\begin{array}{c} X_{3D}=Concat(H_{0},H_{1},H_{2},H_{5}) \end{array}$$

where $X_{1D}$, $X_{2D}$, and $X_{3D}$ denote the feature maps extracted from the 1D, 2D, and 3D branches, respectively. $G_{d}$, $F_{d}$, and $H_{d}$ represent the outputs of the 1D, 2D, and 3D convolutions with dilation rates d=[0,1,2,5], respectively.

### 2.3 Linear-projection feature pyramid module

The core idea of the Linear-Projection Feature Pyramid Module (LPFPM) is to capture spatial features at multiple scales through depthwise convolutions (DC) and to fuse these features to enhance the model’s ability to perceive multi-scale information. Specifically, the multi-dimensional feature maps $X_{1D}$, $X_{2D}$, and $X_{3D}$ produced by the MDFEM are first concatenated to obtain the fused representation $X_{\text{fusion}}$. The formulation is given in Equation 6:

(6) $$\begin{array}{c} X_{fusion}=Concat(X_{1D},X_{2D},X_{3D}) \end{array}$$

Next, $X_{\text{fusion}}$ is passed through a linear projection layer to reduce its feature dimensionality, yielding $X_{fusion}^{\prime }$. This dimensionality reduction step effectively decreases the computational cost of the subsequent convolutional operations and improves overall efficiency. The process is formulated in Equation 7.

(7) $$X_{fusion}^{\prime }=Linear(X_{fusion})$$

**Figure 2:**
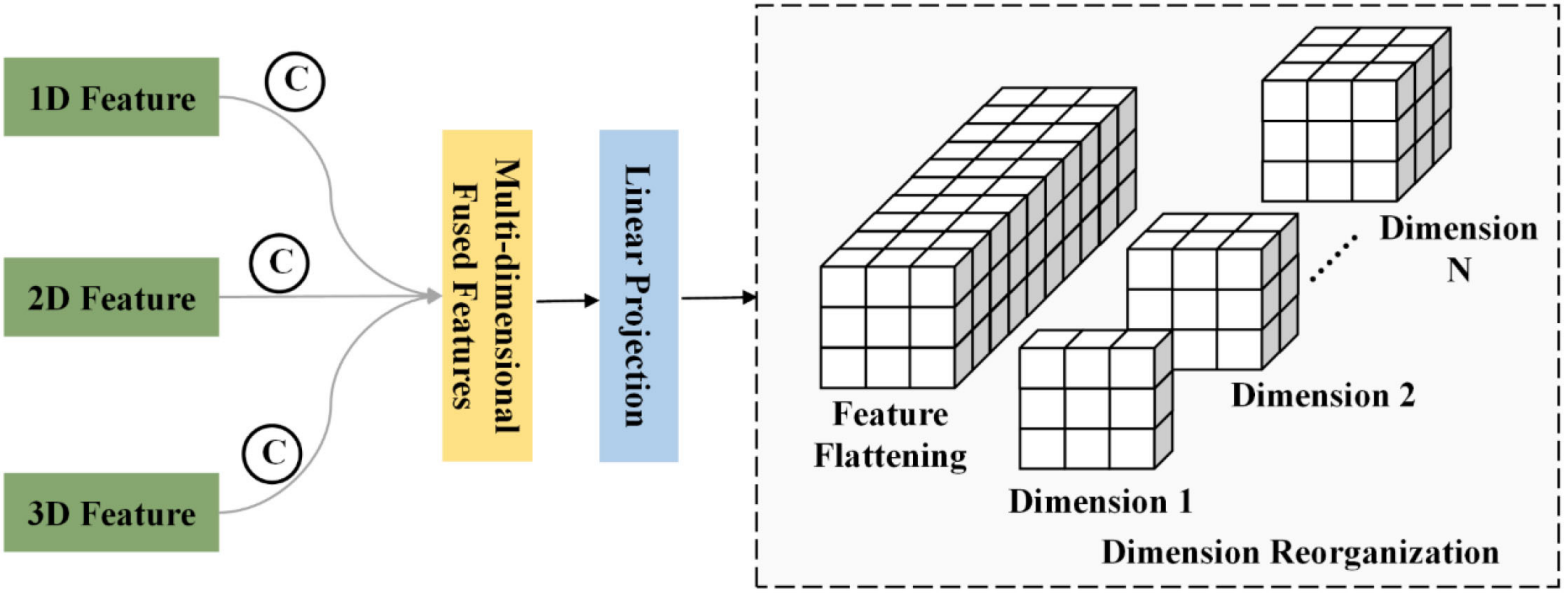
Feature fusion and reorganization.

Subsequently, the reduced feature representation is flattened and partitioned into N groups along the channel dimension, forming N feature maps with different dimensional configurations. Each group is processed by convolutional layers of different scales, as illustrated in **Figure 2**. This operation is formulated in Equation 8:

(8) $$X_{11}^{\prime },X_{22}^{\prime },\ldots \ldots ,X_{NN}^{\prime }=Split(X_{fusion}^{\prime })$$

where $X_{1}^{\prime },X_{2}^{\prime },\ldots ,X_{N}^{\prime }$ denote the N groups of feature vectors obtained after channel-wise partitioning.

**Figure 3:**
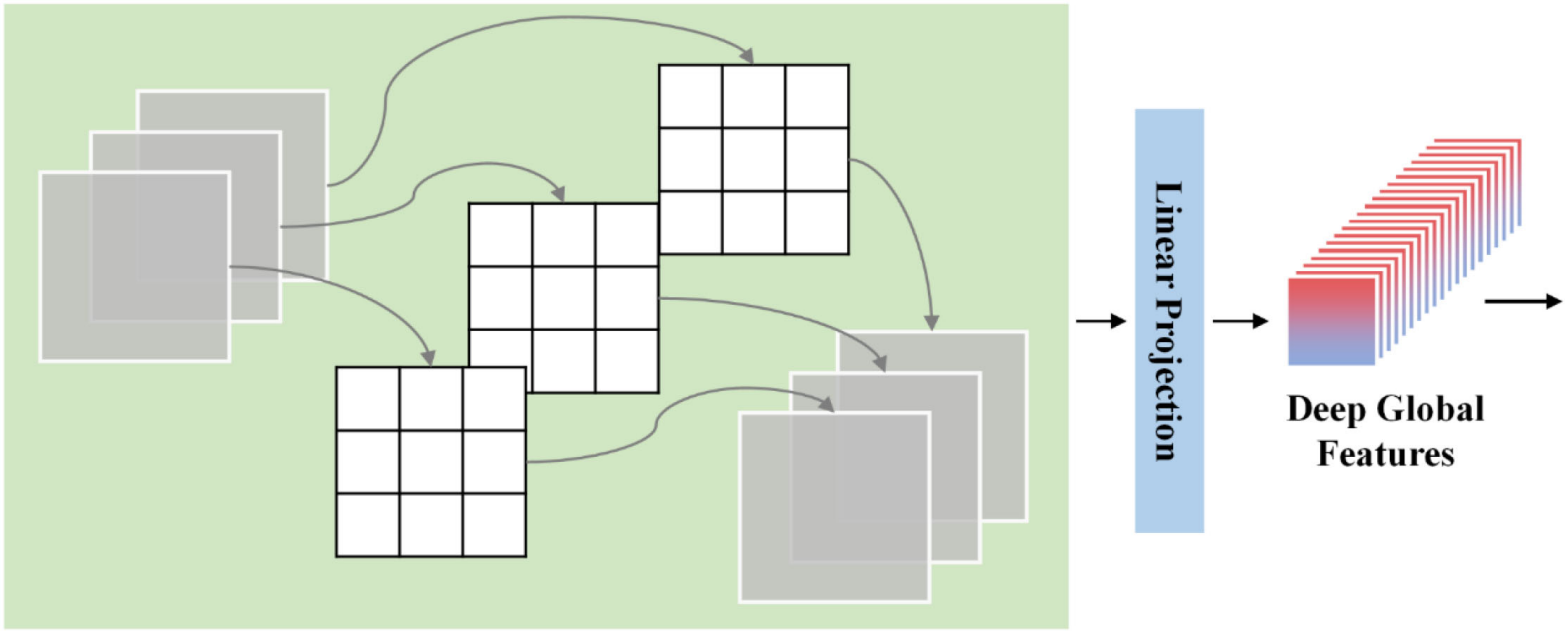
Depthwise pointwise convolution.

Finally, depthwise pointwise convolution (DPC) is applied to the feature maps of different dimensional groups. Depthwise convolution is a special form of grouped convolution in which the number of groups is equal to the number of input channels, meaning that each channel is processed independently with its own convolution kernel while the number of output channels remains unchanged (**Figure 3**). Since no cross-channel interaction is involved and each channel uses a separate filter, this decomposition significantly reduces the number of parameters and computational cost.

Assuming that the input feature map is $X\in {\mathbb{R}}^{H\times W\times C_{in}}$, a standard convolution with a kernel size of K×K and $C_{out}$ output channels results in a computational cost $\omega_{s}$, as defined in Equation 9. In comparison, the computational cost of depthwise pointwise convolution (DPC) is denoted as $\omega_{DPC}$, as shown in Equation 10.

(9) $$\begin{array}{c} \omega_{s}=K\times K\times \;H\times W\times C_{in}\times C_{out} \end{array}$$

(10) $$\begin{array}{c} \omega_{DPC}=(K+C_{out})\times K\times \;H\times W\times C_{in} \end{array}$$

After applying DPC, the reduction in the number of parameters, denoted as $\omega_{\text{Δ}}$, is calculated as shown in Equation 11.

(11) $$\begin{array}{c} \omega_{\text{Δ}}=\frac{(K\times K+C_{out})\times C_{in}}{K\times K\times C_{in}\times C_{out}}=\frac{1}{C_{out}}+\frac{1}{K^{2}} \end{array}$$

### 2.4 Transformer encoder

**Figure 4:**
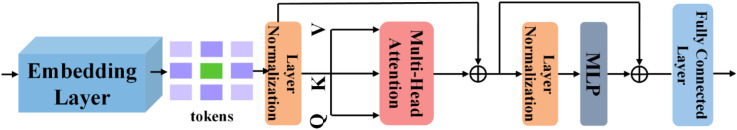
Transformer architecture.

The Transformer encoder consists of an embedding layer, a multi-head self-attention (MHSA) mechanism, and a multi-layer perceptron (MLP), as illustrated in **Figure 4**. First, the deep global features extracted by the LPFPM are transformed into a sequence of tokens that can be processed by the Transformer architecture. The tokens are then passed through a layer normalization operation to stabilize the feature distribution prior to attention computation. The encoder follows a standard Transformer architecture, configured with a token dimension of 64, a depth of 1 layer, 8 attention heads, an MLP hidden dimension of 8, and a dropout rate of 0.1.

First, the spectral-spatial features extracted by the parallel 2D, 1D, and 3D convolutional branches are fused by the Multi-scale Receptive Field Fusion (MRFP) module. The MRFP employs three parallel depth-wise convolutions at different scales (original, downsampled, and upsampled) to capture multi-contextual information, followed by feature concatenation and a pointwise convolution for fusion. These fused deep global features are then projected and transformed into a sequence of tokens for processing by the Transformer. Specifically, the tokenization process is implemented via two sets of learnable projection matrices, which compress the spatial-spectral feature sequence into a fixed number of 4 tokens, each with a dimension of 64. The tokens, along with a prepended learnable classification token, are subsequently passed through a layer normalization operation to stabilize the feature distribution prior to attention computation.

In the MHSA module, each pixel-level feature vector is projected into three independent subspaces—query (Q), key (K), and value (V)—allowing the model to capture feature relationships across different representation subspaces. The outputs from multiple attention heads are then concatenated and linearly transformed to project them back into the output space, which reduces dimensionality while retaining essential information. The similarity between the query and key vectors is computed as shown in Equation 12:

(12) $$\begin{array}{c} MHSA(X)=Attention(Q,K,V)=Softmax(\frac{QK^{T}}{\sqrt{d_{k}}})V \end{array}$$

The dot-product operation between Q and $K^{T}$ reflects the similarity between the two vectors. To obtain normalized attention weights, a softmax function is applied, converting the similarity matrix into an attention weight matrix. The resulting attention weights are then multiplied with the value vectors V, enabling each token to attend to other tokens and capture complex relationships such as “different spectra within the same region” and “similar spectra across different regions,” thereby uncovering intrinsic spectral variations within crops.

The MLP is a fundamental feed-forward neural network that ensures unidirectional information flow. Data enter from the input layer, are processed through one or more hidden layers, and finally pass to the output layer, with no feedback connections between layers. The output of the MLP layer is formulated in Equation 13:

(13) $$\begin{array}{c} \text{M}\text{L}\text{P}(X)=\tau (0,\text{X}W_{1}+b_{1})W_{2}+b_{2} \end{array}$$

where $W_{1}$, $b_{1}$ and $W_{2}$, $b_{2}$ represent the weights and biases of the two linear transformations, respectively, and τ denotes the nonlinear activation function. To mitigate covariate shift and provide more stable gradient signals, normalization layers are introduced before both the MHSA and MLP blocks.

## 3 Data sources

**Figure 5:**
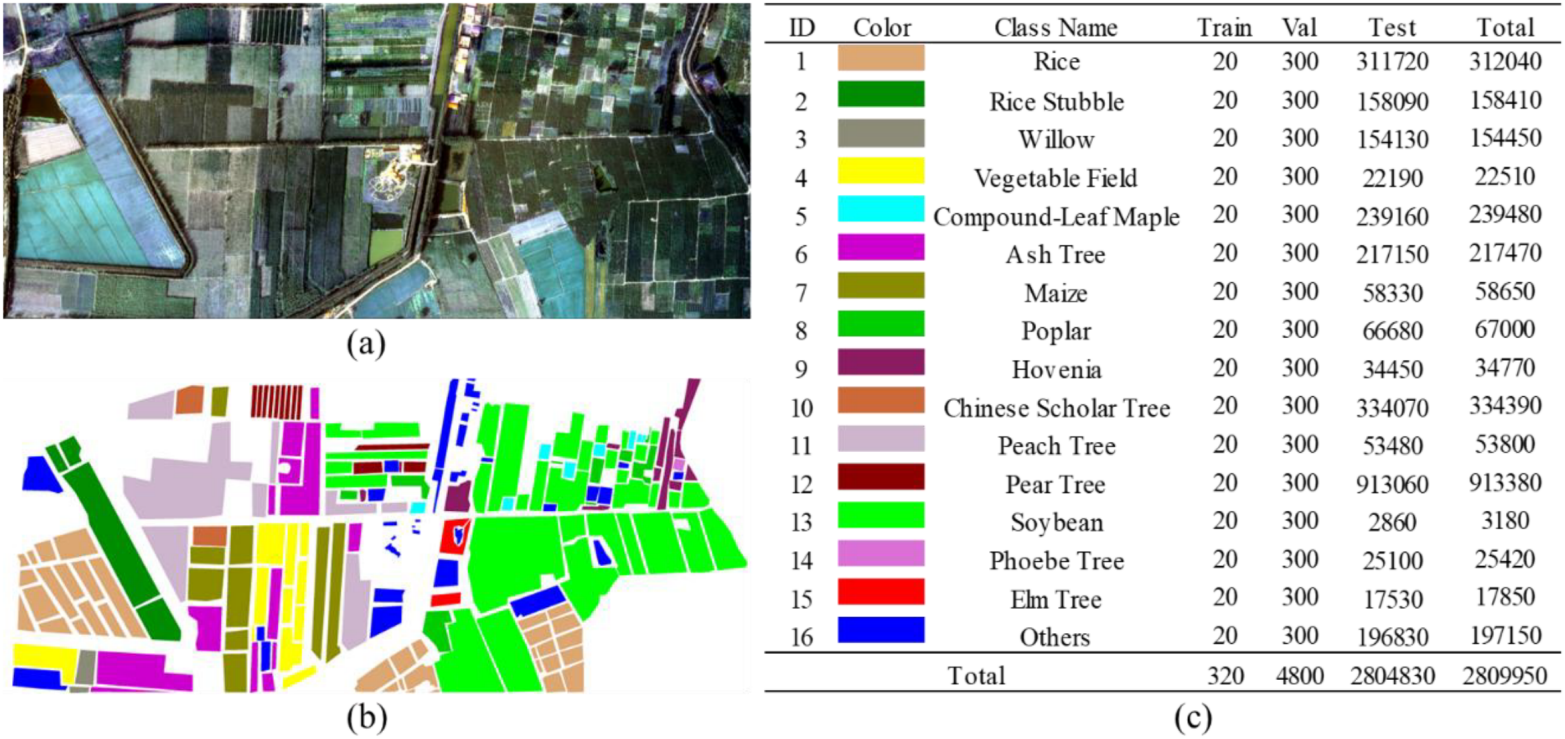
Original image and sample labels of the matiwan village dataset. **(a)** Original image. **(b)** Sample labels. **(c)** Color mapping of categories and pixel counts of training, validation, and test sets.

**Figure 6:**
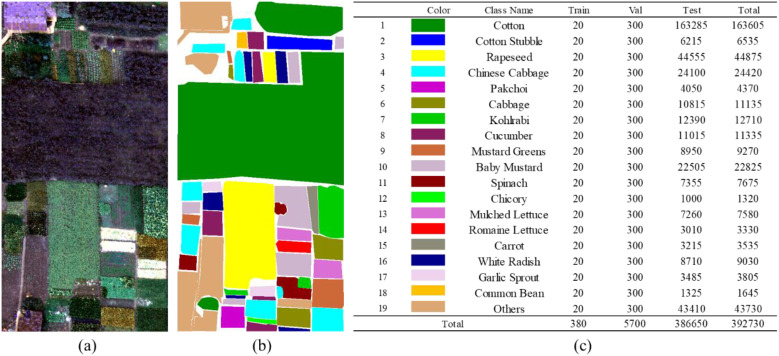
Original image and sample labels of the WHU-HongHu dataset. **(a)** Original image. **(b)** Sample labels. **(c)** Color mapping of categories and pixel counts of training, validation, and test sets.

**Figure 7:**
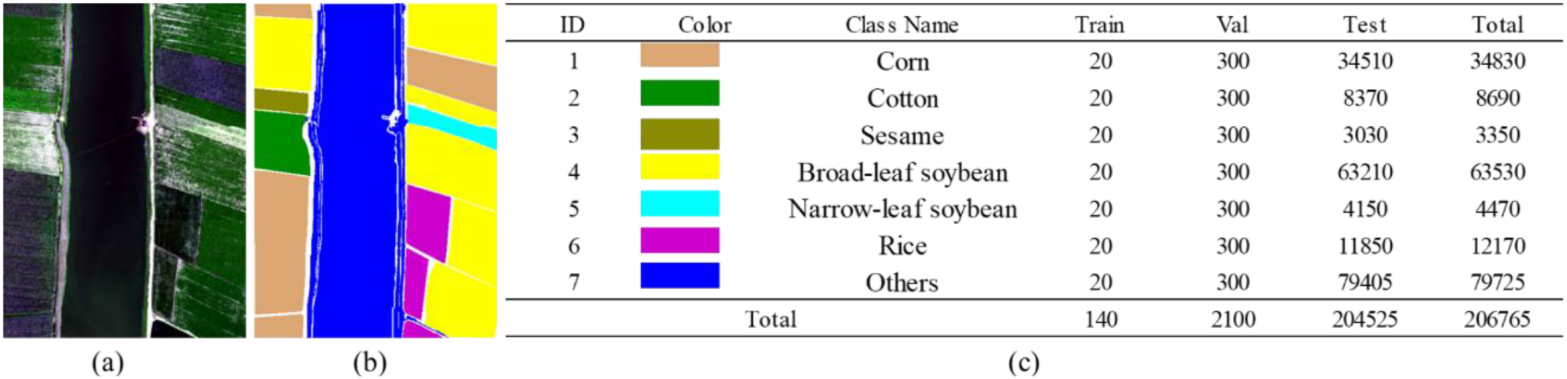
Original image and sample labels of the WHU-LongKou dataset. **(a)** Original image. **(b)** Sample labels. **(c)** Color mapping of categories and pixel counts of training, validation, and test sets.

In this section, we introduce the three hyperspectral datasets used in our experiments. The original HSI images and the corresponding ground-truth label maps are illustrated in **Figures 5**–**7**. For all datasets, non-crop categories are uniformly assigned to an “other” class.

### 3.1 Matiwan Village dataset

The first dataset was acquired from aerial hyperspectral imagery collected in Matiwan Village, Xiong’an New Area, Baoding City, Hebei Province, China. The dataset contains 256 spectral bands covering a wavelength range of 400–1000 nm, with a spectral resolution of 2.1 nm. The spatial resolution of the imagery is 0.5 m, and the scene consists of 3750 × 1580 pixels. A total of 18 land-cover categories are present in the dataset, among which 16 crop types are selected as the target classes for this study, while the remaining categories are grouped into an “other” class.

### 3.2 WHU-HongHu dataset

The second dataset was collected on November 20, 2017, in Honghu City, Hubei Province, China. During data acquisition, parts of the UAV-based hyperspectral imagery were slightly affected by cloud occlusion. The dataset covers a spatial extent of 940 × 475 pixels with a spatial resolution of 0.043 m and contains 270 spectral bands. It consists of a variety of complex crop types, with certain categories (e.g., Chinese cabbage and bok choy) exhibiting high spectral similarity. A total of 22 land-cover classes are included in the dataset, among which 19 crop types are selected as the target categories for this study, while the remaining classes are grouped into an “other” category.

### 3.3 WHU-LongKou dataset

The third dataset was acquired on July 17, 2018, using UAV-based hyperspectral imaging in Longkou Town, Hubei Province, China. Weather conditions during acquisition were clear, with no cloud or rainfall interference. The dataset has a spatial size of 400 × 550 pixels with an approximate spatial resolution of 0.463 m. It contains 270 spectral bands spanning a wavelength range of 400–1000 nm. A total of nine land-cover categories are present in the dataset, among which seven crop types are selected as the target classes for this study, while the remaining categories are assigned to an “other” class.

## 4 Experimental settings and results

### 4.1 Experimental settings

(1) Parameter settings: To ensure fairness and reproducibility, all experiments were conducted on a workstation equipped with an Intel(R) Core(TM) i9-14900KF CPU, an NVIDIA GeForce RTX 5090 GPU, and 128 GB RAM. All models were implemented and trained using the PyTorch deep learning framework. The number of training epochs was set to 500. Each experiment was repeated five times, and the average performance was reported to improve the reliability of the results.

(2) Patch size configuration: Considering the trade-off among computational cost, classification accuracy, and the ability to capture both local details and contextual information within hyperspectral imagery, an input patch size of 11×11 was selected for all experiments.

(3) Sample proportion settings: For the WHU-LongKou and WHU-HongHu datasets, an equal number of training samples was adopted for fairness. Specifically, five random groups of samples were selected, with each group containing 20 pixels for training and 300 pixels for validation, while the remaining pixels were used as the test set. For the Matiwan Village dataset, five random groups were also selected, each containing 100 pixels for training and 300 pixels for validation, with the remaining pixels used for testing. This setting allows us to evaluate the model’s performance under balanced limited sample scenarios.

(4) Learning rate settings: In this study, five learning rates— $1\times 10^{-3}$, $1\times 10^{-4}$, $5\times 10^{-4}$, $1\times 10^{-5}$, and $5\times 10^{-5}$ —were tested on the three datasets. After extensive experimentation, a learning rate of $1\times 10^{-4}$ was determined to be the optimal choice.

(5) Evaluation metrics::Overall accuracy (OA), average accuracy (AA), and the Kappa coefficient are widely used metrics for evaluating classification performance.

OA measures the percentage of correctly classified pixels relative to the total number of pixels in the test set, reflecting the overall classification performance. It is computed as (Equation 14):

(14) $$\begin{array}{c} \text{O}\text{A}=\frac{\sum_{i=1}^{k}{\text{TP}}_{i}}{\text{N}}\times 100\% \end{array}$$

where k denotes the number of crop categories, $TP_{i}$ represents the number of correctly classified pixels for class i, and N is the total number of pixels in the test set.

AA represents the arithmetic mean of the classification accuracy of each class, helping to reduce the influence of class imbalance. It is defined as (Equation 15):

(15) $$\begin{array}{c} \text{A}\text{A}=\frac{1}{\text{k}}\sum_{i=1}^{k}(\frac{{\text{TP}}_{i}}{n_{i}})\times 100\% \end{array}$$

where $n_{i}$ refers to the number of true pixels belonging to class i in the test set.

The Kappa coefficient is an agreement measure that evaluates the consistency between classification results and ground truth while accounting for agreement occurring by chance. A higher Kappa value indicates more reliable classification performance. It is calculated as (Equation 16):

(16) $$\begin{array}{c} \text{K}\text{a}\text{p}\text{p}\text{a}=\frac{\text{OA}-{\text{p}}_{e}}{1-{\text{p}}_{e}} \end{array}$$

where the expected agreement $p_{e}$ under random classification is given by (Equation 17):

(17) $$\begin{array}{c} p_{e}=\sum_{i=1}^{k}\frac{{\text{n}}_{i}\times {\text{m}}_{i}}{{\text{N}}^{2}} \end{array}$$

Here, $m_{i}$ denotes the number of pixels predicted as class i in the classification results.

### 4.2 Experimental results

We compare the proposed method with six state-of-the-art deep learning–based HSI classification approaches, including conventional CNN-based models such as 3D-CNN (Zunair et al., 2020), However, since the classification accuracy of 3D-CNN for each category lags behind that of other advanced models, it is not included in the comparison table. As well as Transformer-based methods such as SSFTT (Sun et al., 2022), MorphFormer (Roy et al., 2023), GAHT (Mei et al., 2022), GSC-ViT (Zhao et al., 2024), CTMixer (Zhang et al., 2022), and WD-SSMamba (Zhang et al., 2025).

SSFTT captures spectral–spatial representations and higher-level semantic features through a spectral–spatial feature tokenization transformer. MorphFormer is a learnable spectral–spatial morphological network that improves interaction between structural and shape-related information in HSI labeling. GAHT introduces a group-aware pixel embedding module that constrains MHSA within local spectral–spatial contexts. GSC-ViT incorporates a grouped separable convolution (GSC) module that significantly reduces convolutional parameters while effectively capturing local spectral–spatial information. CTMixer adopts a dual-branch architecture combining CNNs and Transformers to jointly extract local and global hyperspectral features. Mamba-based models, including WD-SSMamba, utilize a state-space model architecture, which dynamically captures long-range dependencies while reducing computational complexity through efficient token generation. The Mamba framework facilitates lightweight and effective modeling of spectral and spatial features in hyperspectral image classification tasks.

#### 4.2.1 Matiwan Village dataset

The classification performance of MDPC-Net and the six comparison models on the Matiwan Village dataset is summarized in **Table 1**. MDPC-Net achieves an OA of 88.82%, an AA of 94.20%, and a Kappa coefficient of 0.8702 across 16 crop categories, demonstrating strong overall classification capability under limited-sample conditions. MDPC-Net attains the highest classification accuracy for 15 out of 16 crop types, confirming the effectiveness of the proposed multi-dimensional feature coupling strategy.

**Table 1:** Accuracy evaluation of MDPC-Net vs. six comparative models on the matiwan village dataset.

Label		SSFTT	GAHT	Morphformer	GSC-Vit	CTMixer	WD-SSMamb	MDPC-Net
1	Rice	98.77 ± 0.52	98.48 ± 0.47	98.74 ± 0.53	98.70 ± 0.43	99.36 ± 0.51	99.39 ± 1.52	**99.63 ± 0.47**
2	Rice Stubble	99.43 ± 0.37	99.37 ± 0.50	99.16 ± 0.62	98.93 ± 1.02	99.63 ± 0.33	99.41 ± 1.08	**99.82 ± 0.18**
3	Willow	85.01 ± 1.30	81.84 ± 1.72	79.10 ± 1.78	81.78 ± 1.85	86.56 ± 2.19	96.02 ± 1.65	**96.29 ± 0.57**
4	Vegetable Field	77.17 ± 4.18	76.52 ± 3.42	73.38 ± 1.07	77.37 ± 4.53	78.22 ± 2.54	92.24 ± 1.89	**92.54 ± 2.59**
5	Compound-Leaf Maple	76.78 ± 1.16	73.10 ± 2.04	73.21 ± 2.28	74.94 ± 3.12	81.52 ± 2.05	91.43 ± 1.12	**91.83 ± 1.15**
6	Ash Tree	74.18 ± 2.31	76.50 ± 4.24	76.29 ± 1.68	77.15 ± 3.85	81.14 ± 2.71	90.45 ± 0.74	**90.87 ± 1.43**
7	Maize	74.82 ± 2.75	73.34 ± 4.05	71.50 ± 2.92	70.64 ± 4.11	81.68 ± 2.01	89.82 ± 0.84	**90.01 ± 1.82**
8	Poplar	88.44 ± 1.14	88.29 ± 1.89	87.14 ± 2.00	87.20 ± 0.79	89.33 ± 2.10	94.96 ± 1.20	**95.45 ± 0.56**
9	Hovenia	89.02 ± 2.18	91.61 ± 1.48	90.54 ± 1.45	90.87 ± 0.98	92.96 ± 2.80	98.19 ± 0.63	**98.37 ± 0.58**
10	Chinese Scholar Tree	68.28 ± 2.67	68.25 ± 1.67	66.27 ± 4.78	66.42 ± 3.79	70.11 ± 5.26	87.98 ± 1.92	**88.29 ± 1.21**
11	Peach Tree	87.65 ± 2.10	88.09 ± 2.99	86.10 ± 2.81	88.94 ± 3.63	89.35 ± 1.64	97.05 ± 1.15	**97.17 ± 0.87**
12	Pear Tree	62.14 ± 1.65	59.46 ± 2.81	57.38 ± 2.87	59.85 ± 7.02	62.46 ± 3.61	78.35 ± 0.55	**78.50 ± 2.00**
13	Soybean	99.44 ± 0.78	98.74 ± 0.78	99.02 ± 0.26	99.09 ± 0.52	99.51 ± 0.52	99.47 ± 1.64	**99.86 ± 0.17**
14	Phoebe Tree	96.77 ± 0.50	96.64 ± 1.15	96.72 ± 0.69	97.49 ± 0.80	96.65 ± 0.52	98.5 ± 0.70	**98.64 ± 0.24**
15	Elm Tree	96.24 ± 0.39	96.06 ± 1.27	94.10 ± 1.19	94.28 ± 0.88	96.97 ± 1.06	98.97 ± 0.66	**99.41 ± 0.41**
16	Others	84.99 ± 1.32	83.70 ± 1.26	84.85 ± 1.34	84.60 ± 1.20	86.52 ± 1.19	90.2 ± 1.04	**90.49 ± 1.64**
OA		76.47 ± 0.36	75.16 ± 1.28	74.03 ± 1.04	78.26 ± 1.76	75.26 ± 3.14	**88.66 ± 1.29**	**88.82 ± 0.75**
AA		84.95 ± 0.19	84.38 ± 0.83	83.34 ± 0.73	87.00 ± 0.69	84.26 ± 1.32	**93.86 ± 0.52**	**94.20 ± 0.26**
K×100		73.11 ± 0.38	71.64 ± 1.39	70.46 ± 1.12	75.12 ± 1.91	71.80 ± 3.35	**86.77 ± 0.83**	**87.02 ± 0.84**

The bold values indicate the highest accuracy results among the compared methods for each corresponding metric.

**Figure 8:**
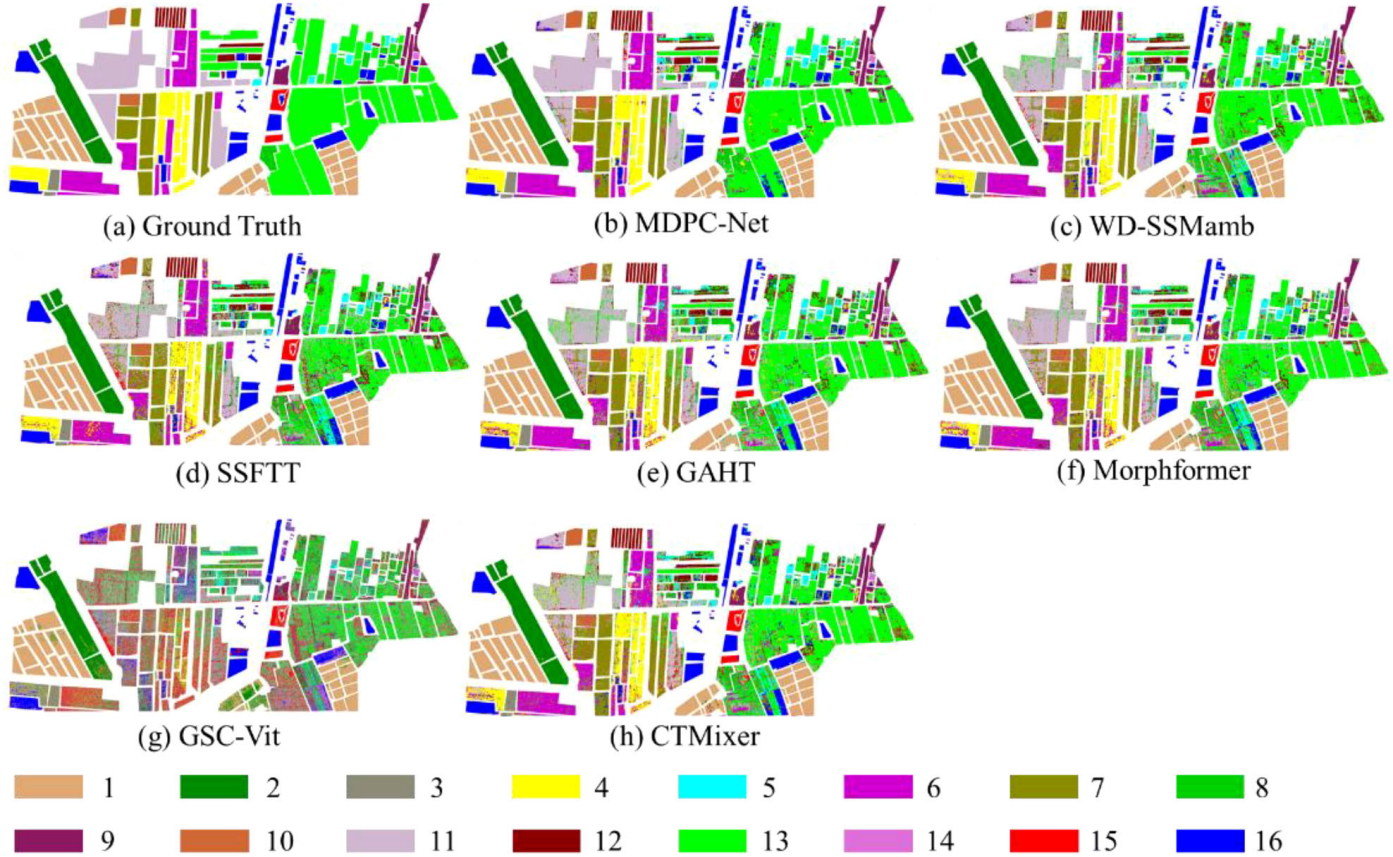
Sample classification results of different models on the matiwan village dataset. The numerical labels and their corresponding color patches indicate distinct crop categories, with the specific crop type for each label detailed in **Figure 5**.

Nevertheless, a small number of spectrally similar classes still exhibit relatively lower classification accuracy. For instance, the accuracies for pear tree and Chinese scholar tree are 78.50% and 88.29%, respectively. This performance degradation can be primarily attributed to substantial spectral overlap between these vegetation types, which share similar canopy structures, biochemical compositions, and phenological characteristics in the Matiwan Village region. Under such conditions, even high-dimensional hyperspectral features provide limited inter-class separability, making precise discrimination inherently challenging. Although MDPC-Net still outperforms most comparison models on these classes, the remaining performance gap highlights an intrinsic limitation imposed by spectral similarity rather than model insufficiency. From a methodological perspective, this observation suggests that classification performance for highly spectrally similar crops may benefit from incorporating additional discriminative cues, such as multi-temporal information, fine-grained texture descriptors, or domain-specific prior knowledge (e.g., phenological constraints). Future extensions of the proposed framework could explore temporal-aware feature modeling or adaptive class-specific refinement mechanisms to further enhance discrimination among such challenging categories. The qualitative classification results are illustrated in **Figure 8**. Compared with the ground-truth map (**Figure 8a**), methods such as WD-SSMamba (**Figure 8c**) and SSFTT (**Figure 8d**) tend to generate fragmented predictions in large homogeneous regions, erroneously splitting continuous crop fields into multiple categories. This phenomenon indicates limited robustness in maintaining spatial consistency, particularly under complex agricultural layouts. GSC-ViT (**Figure 8g**) better captures large-scale structural patterns but still suffers from noticeable loss of local details, especially near field boundaries.

In contrast, MDPC-Net (**Figure 8b**) demonstrates superior spatial coherence and structural fidelity. Its predictions exhibit strong alignment with the ground truth across diverse spatial patterns, including large continuous green regions, regular purple and brown block-like fields, and smaller scattered blue and red patches. This improvement can be attributed to the proposed multi-dimensional progressive feature extraction and linear-projection pyramid fusion, which jointly preserve both global contextual semantics and fine-grained local structures.

Furthermore, Mamba-based models, such as WD-SSMamba (**Figure 8c**), show noticeable improvements over conventional CNN- and Transformer-based approaches by more effectively modeling long-range dependencies while maintaining computational efficiency. However, compared with MDPC-Net, their spatial predictions still exhibit occasional inconsistencies in boundary delineation and local detail preservation. This comparison highlights that while Mamba-based architectures provide an efficient mechanism for global dependency modeling, the explicit multi-dimensional feature coupling and hierarchical fusion strategy employed by MDPC-Net is more effective in addressing the complex spectral–spatial characteristics of agricultural scenes.

#### 4.2.2 WHU-HongHu dataset

The classification performance on the WHU-HongHu dataset is summarized in **Table 2**. MDPC-Net achieves an OA of 89.95%, an AA of 89.63%, and a Kappa coefficient of 0.8730 across 18 crop categories, demonstrating strong overall performance on this large-scale and fine-grained agricultural dataset. MDPC-Net outperforms the six comparison methods for most crop types, confirming its robustness under complex class distributions and limited training samples.

**Table 2:** Accuracy evaluation of MDPC-Net versus six comparative models on the WHU-HongHu dataset.

Label		SSFTT	GAHT	Morphformer	GSC-Vit	CTMixer	WD-SSMamba	MDPC-Net
1	Cotton	93.55 ± 2.00	93.32 ± 1.86	95.67 ± 1.20	95.68 ± 1.69	**96.75 ± 1.42**	94.94 ± 1.42	96.24 ± 1.42
2	Cotton Stubble	91.54 ± 2.95	90.43 ± 2.73	92.73 ± 3.86	92.18 ± 3.06	92.28 ± 1.42	95.09 ± 1.8	**95.38 ± 1.80**
3	Rapeseed	91.44 ± 1.85	87.64 ± 2.96	89.79 ± 3.13	88.93 ± 1.50	90.73 ± 2.34	93.96 ± 1.67	**94.12 ± 3.12**
4	Chinese Cabbage	66.10 ± 5.30	61.54 ± 4.91	61.99 ± 2.90	67.10 ± 3.97	66.70 ± 2.57	72.85 ± 1.38	**73.09 ± 3.65**
5	Pakchoi	68.72 ± 3.09	57.23 ± 7.23	67.85 ± 10.04	64.05 ± 6.77	68.20 ± 5.79	**80.46 ± 0.67**	80.27 ± 3.86
6	Cabbage	97.23 ± 1.51	94.29 ± 2.57	97.41 ± 0.40	95.59 ± 1.70	97.25 ± 1.15	97.53 ± 1.0	**97.89 ± 1.02**
7	Kohlrabi	85.55 ± 3.12	73.83 ± 4.55	84.16 ± 6.87	78.18 ± 3.61	81.19 ± 2.88	87.40 ± 1.54	**87.70 ± 3.65**
8	Cucumber	69.61 ± 4.29	62.71 ± 4.51	66.54 ± 6.25	70.78 ± 5.38	72.29 ± 5.08	80.21 ± 1.89	**80.37 ± 4.62**
9	Mustard Greens	74.27 ± 4.62	70.36 ± 7.12	74.11 ± 4.86	75.62 ± 5.61	73.58 ± 2.97	77.42 ± 1.06	**77.56 ± 5.18**
10	Baby Mustard	67.55 ± 5.04	61.71 ± 5.88	66.78 ± 6.48	67.15 ± 3.30	63.53 ± 4.72	71.23 ± 1.17	**72.76 ± 4.58**
11	Spinach	90.09 ± 2.71	77.24 ± 4.75	88.17 ± 0.90	81.52 ± 4.81	87.00 ± 1.80	93.38 ± 0.72	**93.58 ± 1.22**
12	Chicory	97.30 ± 1.72	95.50 ± 4.15	95.90 ± 3.53	94.80 ± 3.04	96.60 ± 2.52	96.48 ± 1.56	**98.70 ± 1.08**
13	Mulched Lettuce	92.07 ± 3.00	89.71 ± 3.61	92.44 ± 3.90	90.45 ± 2.45	93.06 ± 2.06	93.73 ± 1.2	**94.44 ± 2.81**
14	Romaine Lettuce	92.66 ± 3.47	92.99 ± 3.58	95.32 ± 3.00	94.52 ± 3.99	95.15 ± 2.75	96.99 ± 1.39	**97.38 ± 2.45**
15	Carrot	92.53 ± 2.19	87.34 ± 3.91	91.63 ± 3.47	92.72 ± 2.67	93.16 ± 3.32	96.15 ± 1.33	**96.36 ± 1.32**
16	White Radish	90.36 ± 2.34	83.04 ± 4.73	85.15 ± 2.20	86.06 ± 4.11	87.99 ± 4.38	91.38 ± 0.55	**91.49 ± 3.89**
17	Garlic Sprout	93.46 ± 1.91	94.09 ± 3.01	93.17 ± 2.97	93.92 ± 2.31	96.76 ± 2.20	97.07 ± 1.09	**97.36 ± 0.93**
18	Common Bean	96.08 ± 2.41	90.49 ± 6.98	95.70 ± 3.19	94.11 ± 2.20	94.49 ± 4.48	97.12 ± 1.21	**97.89 ± 1.18**
19	Others	75.57 ± 1.87	76.66 ± 3.28	78.20 ± 3.48	77.46 ± 1.71	77.57 ± 2.99	78.94 ± 1.55	**80.35 ± 2.50**
OA		86.33 ± 1.11	83.89 ± 1.17	86.76 ± 1.33	86.65 ± 1.12	87.50 ± 0.70	88.98 ± 1.48	**89.95 ± 0.90**
AA		85.56 ± 0.85	81.06 ± 0.95	84.88 ± 1.71	84.25 ± 1.02	85.49 ± 0.46	88.99 ± 0.88	**89.63 ± 0.76**
K×100		82.84 ± 1.32	79.82 ± 1.39	83.32 ± 1.64	83.19 ± 1.35	84.21 ± 0.83	86.83 ± 0.51	**87.30 ± 1.09**

The bold values indicate the highest accuracy results among the compared methods for each corresponding metric.

However, several spectrally and morphologically similar categories remain challenging to distinguish. For example, the classification accuracy for cotton is slightly lower than that of CTMixer, whereas cotton residues achieve higher accuracy than CTMixer. This phenomenon is mainly attributed to the high spectral similarity and transitional characteristics between cotton and cotton residues, especially during post-harvest stages, where residual vegetation and soil background introduce mixed spectral responses. Although MDPC-Net effectively leverages multi-dimensional contextual information, such subtle intra-class variability and inter-class overlap inherently limit discriminability under a single-date hyperspectral setting. Similarly, confusion is observed between Chinese cabbage and baby mustard choy, as well as mustard and small mustard, which exhibit closely related spectral signatures and comparable canopy structures. These crop types often share similar biochemical compositions and growth stages, leading to overlapping spectral–spatial representations. While MDPC-Net still outperforms several baseline methods for these classes, the reduced performance relative to other crop categories highlights intrinsic classification difficulty driven by semantic proximity, rather than deficiencies in the proposed framework. From a methodological perspective, these observations suggest that further performance improvements for such confusing classes may require additional discriminative cues beyond single-scene spectral–spatial features. Potential extensions include incorporating multi-temporal hyperspectral observations to exploit phenological differences, integrating object-level structural priors, or introducing class-adaptive feature refinement strategies that emphasize subtle intra-class variations. These directions could further enhance discrimination among crops with high spectral and morphological similarity.

**Figure 9:**
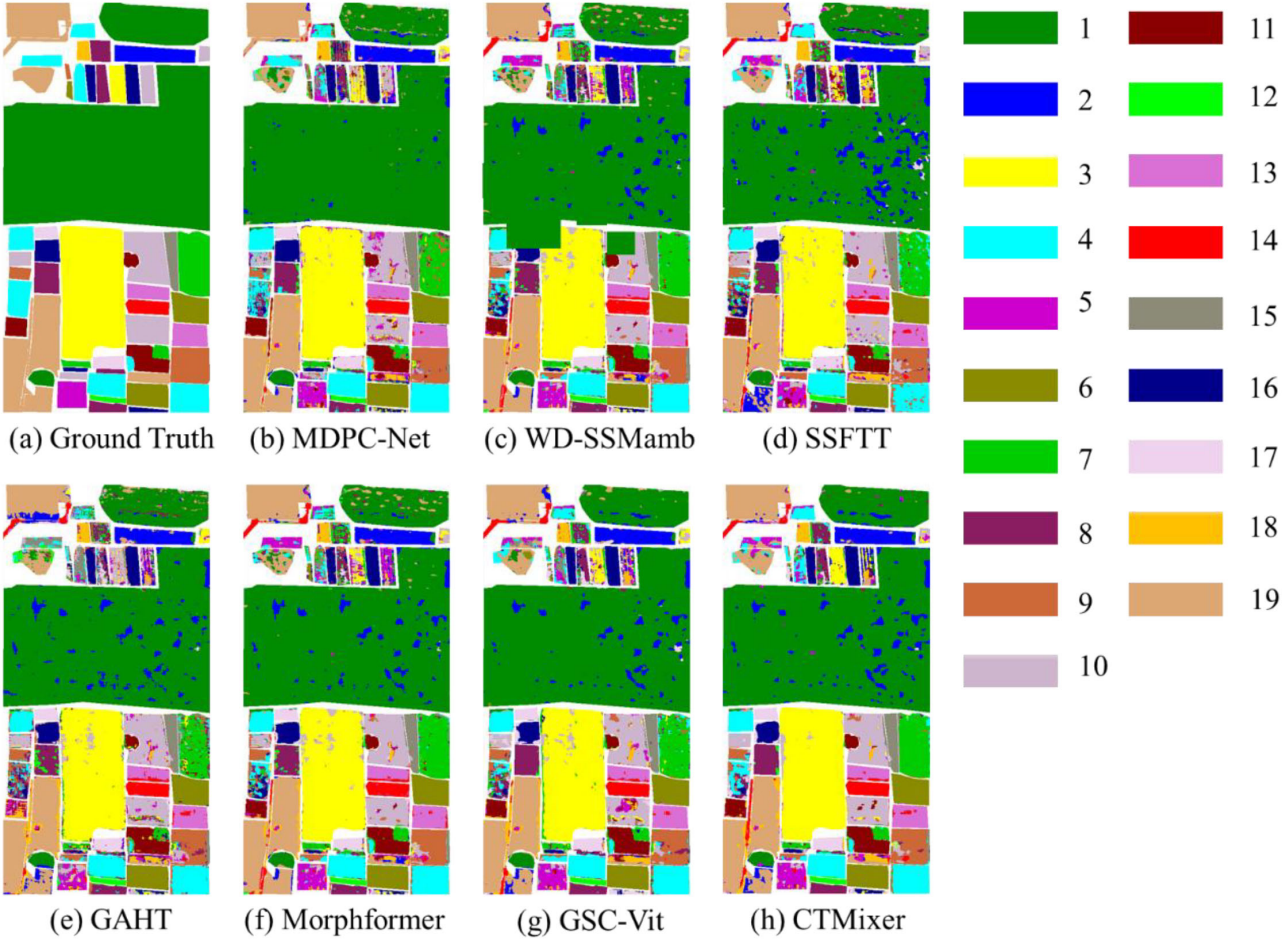
Sample classification results of different models on the WHU-HongHu dataset. The numerical labels and their corresponding color patches indicate distinct crop categories, with the specific crop type for each label detailed in **Figure 6**.

The qualitative classification results are illustrated in **Figure 9**. Compared with the ground-truth map (**Figure 9a**), MDPC-Net (**Figure 9b**) consistently produces classification maps with higher spatial coherence and visual fidelity than all comparison models. Among the Mamba-based methods, such as WD-SSMamba (**Figure 9c**), improved modeling of structural patterns and spatial relationships can be observed when compared with traditional CNN-based approaches. Nevertheless, Mamba-based models still fall short of MDPC-Net in terms of fine-grained detail preservation and boundary accuracy.

Specifically, while WD-SSMamba exhibits clearer region continuity and reduced noise, it still shows limitations in accurately delineating complex field boundaries and small, irregular plots. In contrast, MDPC-Net achieves more precise class transitions at field boundaries and maintains high correspondence with fine-scale ground-truth structures. This advantage is primarily attributed to the LPFPM module, which constructs a multi-scale feature pyramid through multi-dimensional residual connections and linear projections. By effectively integrating spectral, spatial, and spectral–spatial features across scales, LPFPM enhances the model’s adaptability to the heterogeneous and multi-scale characteristics inherent in real-world agricultural landscapes.

#### 4.2.3 WHU-LongKou dataset

The accuracy evaluation of MDPC-Net and six competing models on the WHU-LongKou dataset is presented in **Table 3**. MDPC-Net achieves an OA of 95.70%, an AA of 97.22%, and a Kappa coefficient of 0.9411, indicating an overall high level of classification performance on this dataset. Among the six crop types, MDPC-Net yields slightly lower accuracy than SSFTT only for cotton, while achieving superior or comparable performance for the remaining categories.

**Table 3:** Accuracy evaluation of MDPC-Net versus six comparative models on the WHU-LongKou dataset.

Label		SSFTT	GAHT	Morphformer	GSC-Vit	CTMixer	WD-SSMamb	MDPC-Net
1	Corn	98.66 ± 0.59	98.21 ± 0.61	96.97 ± 2.01	98.42 ± 1.11	99.30 ± 0.28	99.03 ± 1.29	**99.52 ± 0.30**
2	Cotton	98.58 ± 0.61	89.65 ± 5.76	96.63 ± 2.83	95.07 ± 2.16	95.79 ± 2.60	**98.95 ± 1.67**	97.29 ± 1.10
3	Sesame	97.66 ± 0.70	96.30 ± 0.90	94.55 ± 4.09	98.55 ± 0.69	98.25 ± 0.71	99.12 ± 0.87	**99.34 ± 0.47**
4	Broad-leaf soybean	91.13 ± 2.20	87.07 ± 3.29	87.32 ± 3.05	87.03 ± 2.71	90.38 ± 2.14	99.25 ± 1.45	**92.49 ± 1.87**
5	Narrow-leaf soybean	94.07 ± 2.48	89.59 ± 5.28	92.67 ± 4.48	94.51 ± 3.72	94.36 ± 1.46	95.24 ± 1.76	**96.51 ± 1.48**
6	Rice	98.52 ± 1.45	99.54 ± 0.32	97.80 ± 0.42	99.58 ± 0.22	98.39 ± 1.04	99.18 ± 0.92	**99.77 ± 0.20**
7	Others	92.56 ± 2.04	92.08 ± 2.05	88.69 ± 2.31	92.08 ± 2.32	94.19 ± 1.93	95.37 ± 0.76	**95.64 ± 1.99**
OA		93.84 ± 0.64	91.91 ± 1.50	90.69 ± 2.27	92.29 ± 1.33	94.25 ± 0.30	94.72 ± 0.87	**95.70 ± 0.87**
AA		95.88 ± 0.54	93.21 ± 1.62	93.52 ± 2.43	95.03 ± 0.47	95.81 ± 0.45	95.84 ± 0.56	**97.22 ± 0.27**
K×100		91.63 ± 0.84	89.07 ± 1.94	87.50 ± 2.95	89.58 ± 1.73	92.15 ± 0.41	93.54 ± 0.76	**94.11 ± 1.17**

The bold values indicate the highest accuracy results among the compared methods for each corresponding metric.

In addition, the classification accuracies for broad-leaf soybean and narrow-leaf soybean are relatively lower than those of other classes. This behavior can be primarily attributed to the strong spectral similarity and morphological resemblance between these two soybean varieties, which often share overlapping spectral signatures due to similar biochemical composition and growth conditions. Although these classes are spectrally distinguishable from other crops, their intra-group confusion poses a significant challenge for fine-grained classification. Notably, compared with other competing models, MDPC-Net demonstrates a markedly improved capability to discriminate between these two confusing categories, suggesting that the proposed multi-dimensional feature coupling strategy enhances sensitivity to subtle spectral–spatial variations. From a broader perspective, the remaining confusion between soybean subtypes reflects an intrinsic limitation of single-date hyperspectral imagery, where spectral similarity dominates class separability. Future extensions of the proposed framework could explore the integration of multi-temporal observations, phenology-aware representations, or class-specific adaptive refinement modules to further improve discrimination among closely related crop varieties. Across all evaluated models, MDPC-Net also exhibits the lowest standard deviation for most classes, indicating that its predictions are more stable and less sensitive to within-class variability, such as the same crop type appearing across different plots or under slightly varying environmental conditions. This stability highlights the robustness of MDPC-Net and suggests that the learned representations generalize well across spatially heterogeneous agricultural parcels.

**Figure 10:**
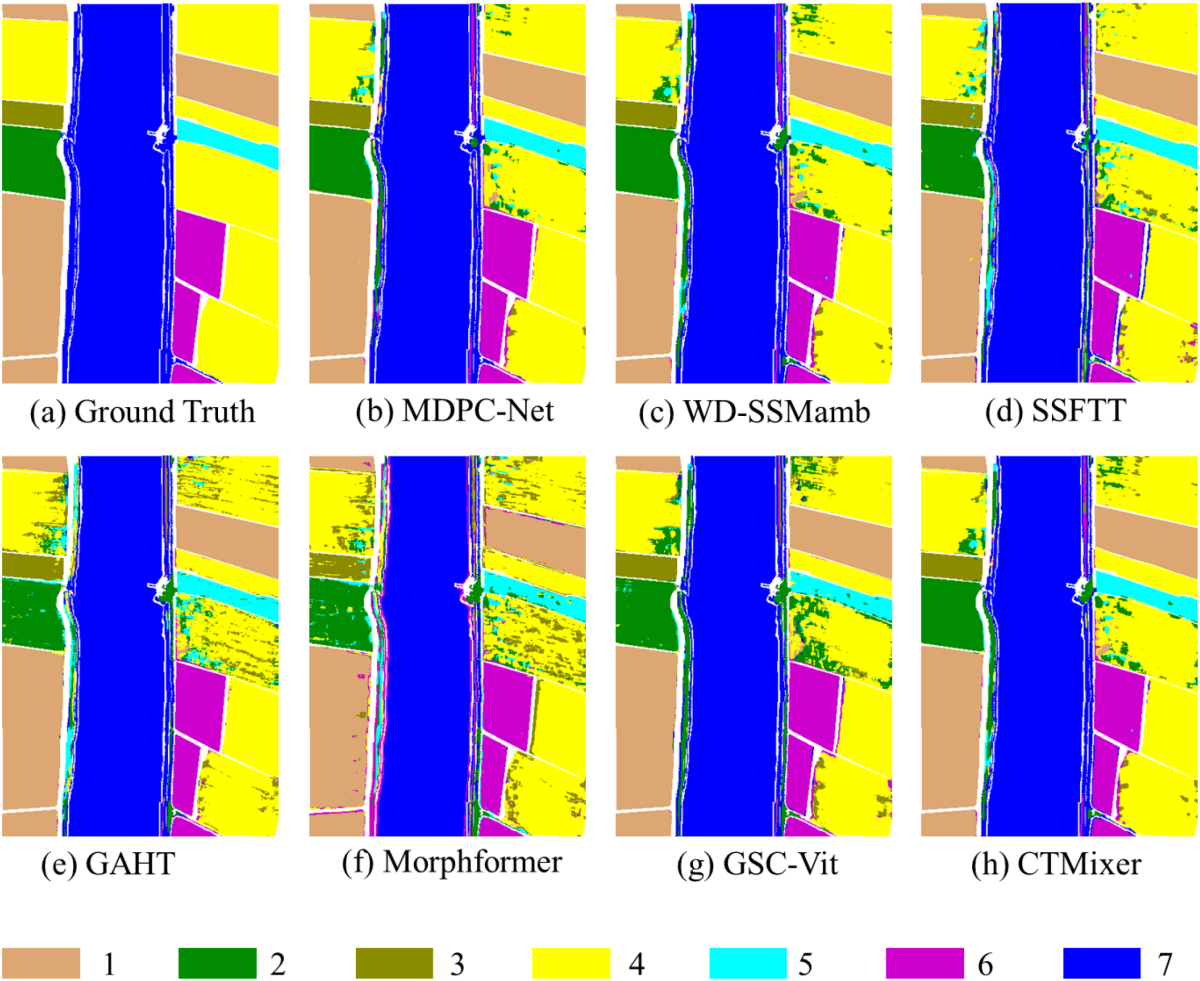
Sample classification results of different models on the WHU-LongKou dataset. The numerical labels and their corresponding color patches indicate distinct crop categories, with the specific crop type for each label detailed in **Figure 7**.

Qualitative comparisons between the ground-truth map (**Figure 10a**) and the classification result produced by MDPC-Net (**Figure 10b**) further support these findings. The overall color distribution and field boundaries are highly consistent with the reference labels. The “others” category exhibits clear and continuous boundaries, while the spatial locations and shapes of corn, cotton, rice, and sesame are accurately preserved, with no evident large-scale misclassification artifacts. Minor errors are mainly observed at the boundaries of broad-leaf soybean fields, where some pixels are misclassified as narrow-leaf soybean or cotton.

In contrast, several competing methods suffer from noticeable loss of detail in small-area classes, particularly cotton, resulting in fragmented or incomplete field representations (**Figures 10c–h**). These results indicate that MDPC-Net more effectively preserves global spatial structure, field morphology, and class distribution consistency, enabling more reliable modeling of crop associations in complex agricultural scenes.

### 4.3 Ablation experiments

To thoroughly validate the effectiveness of the innovative components in our proposed method, we conducted ablation experiments on all three datasets. The ablation study is divided into two parts: Evaluating the contribution of single-branch feature extraction versus the three-branch MDFEM module; and assessing the effectiveness of the LPFPM module.

The results are summarized in **Table 4**. For each dataset, the first row reports the average accuracy obtained from the 1D, 2D, and 3D single-branch extractors combined with MDFEM; the second row presents results using MDFEM alone (without LPFPM); and the third row corresponds to MDFEM combined with LPFPM, i.e., the full MDPC-Net.

**Table 4:** Ablation study of LPFPM versus MDFEM on three datasets.

Datasets	1D/2D/3D	MDFEM	LPFPM	OA	AA	K×100
Matiwan Village	✔		✔	85.82 ± 2.49	92.67 ± 0.88	84.16 ± 2.61
	✔	✔		86.11 ± 1.67	93.25 ± 0.79	85.63 ± 1.78
		✔	✔	**88.82 ± 0.75**	**94.20 ± 0.26**	**87.02 ± 0.84**
WHU-HongHu	✔		✔	84.90 ± 0.47	82.03 ± 1.30	81.00 ± 0.60
	✔	✔		87.50 ± 0.70	85.49 ± 0.46	84.21 ± 0.83
		✔	✔	**89.95 ± 0.90**	**89.63 ± 0.76**	**87.30 ± 1.09**
WHU-LongKou	✔		✔	92.88 ± 1.05	93.89 ± 0.86	90.35 ± 1.37
		✔		93.83 ± 0.67	95.66 ± 0.48	91.61 ± 0.88
		✔	✔	**95.70 ± 0.87**	**97.22 ± 0.27**	**94.11 ± 1.17**

The bold values indicate the highest accuracy results among the compared methods for each corresponding metric.

#### 4.3.1 Individual modules vs. combined modules

Across all ablation configurations and on all three datasets—Matiwan Village, WHU-HongHu, and WHU-LongKou—adding either LPFPM or MDFEM alone consistently improves OA, AA, and Kappa. This demonstrates the independent effectiveness of both modules.

However, the performance improvement achieved by combining MDFEM + LPFPM is substantially greater than the sum of the improvements contributed by each module individually. This indicates that the two modules do not simply provide additive functional benefits; rather, they synergistically enhance feature representation.

Specifically, MDFEM strengthens the discriminability of multi-dimensional features (e.g., through enhanced spectral–spatial fusion), while LPFPM refines the hierarchical transmission within the feature pyramid. When integrated, these two components enable the network to more accurately capture agricultural category variations—such as crop-specific spectral differences and spatial distribution patterns—leading to more precise recognition results.

#### 4.3.2 Robustness across heterogeneous datasets

The three datasets represent varying levels of scene complexity: Matiwan Village corresponds to large heterogeneous farmlands, WHU-HongHu to medium-complex mosaicked fields, and WHU-LongKou to small and relatively simple plots.

In all cases, the combined MDFEM + LPFPM configuration yields consistent and superior improvements in OA, AA, and Kappa compared to individual modules alone (**Table 5**). This demonstrates that the proposed modules effectively adapt to different crop spectral variations and farmland scales across diverse agricultural landscapes.

**Table 5:** Parameters and FLOPs of MDPC-Net on three datasets compared with six models.

Datasets	Methods	Parameters/M	FLOPs/M
Matiwan Village	SSFTT	1.190	62.557
	GAHT	1.498	181.149
	Morphformer	0.244	45.698
	GSC-Vit	0.708	49.073
	CTMixer	0.612	74.314
	WD-SSMamba	**0.153**	**24.667**
	**MDPC-Net**	0.295	33.506
WHU-HongHu	SSFTT	1.255	65.999
	GAHT	1.515	183.100
	Morphformer	0.256	48.206
	GSC-Vit	0.711	49.506
	CTMixer	0.613	74.364
	WD-SSMamba	**0.152**	**24.712**
	**MDPC-Net**	0.306	34.807
WHU-LongKou	SSFTT	1.254	65.998
	GAHT	1.514	183.100
	Morphformer	0.255	48.205
	GSC-Vit	0.711	49.506
	CTMixer	0.612	74.362
	WD-SSMamba	**0.161**	**24.709**
	**MDPC-Net**	0.305	34.806

The bold values indicate the highest accuracy results among the compared methods for each corresponding metric.

The combined architecture therefore exhibits strong robustness to cross-region and cross-scale crop recognition, maintaining reliable performance despite substantial variations in field size, crop type, and scene composition.

### 4.4 Computational complexity analysis

Beyond accuracy, MDPC-Net demonstrates clear advantages in computational efficiency. We computed the number of parameters (Parameters) and floating-point operations (FLOPs) for MDPC-Net, WD-SSMamba, and the six comparison models across all three datasets.

In terms of parameter count, WD-SSMamba contains significantly fewer parameters than MDPC-Net across all datasets. For example, on the Matiwan Village dataset, WD-SSMamba has 0.153M parameters, which is less than half the parameters of MDPC-Net (0.295M parameters). Similarly, WD-SSMamba has 0.152M parameters on WHU-HongHu and 0.161M parameters on WHU-LongKou, both of which are lower than MDPC-Net’s 0.305M parameters. This reduction in parameter count highlights the lightweight design of WD-SSMamba, which is based on advanced wavelet convolutions and the Mamba architecture for efficient feature extraction and representation. Regarding computational cost, MDPC-Net still exhibits a lower FLOPs value than WD-SSMamba in most cases, particularly on the WHU-LongKou dataset, where WD-SSMamba has 24.790M FLOPs, compared to MDPC-Net’s 34.806M FLOPs. While WD-SSMamba benefits from fewer parameters, the complexity of its frequency-domain feature extraction results in higher FLOPs than MDPC-Net in some scenarios. Specifically, on Matiwan Village and WHU-HongHu, WD-SSMamba’s FLOPs (34.667M and 34.712M, respectively) are still lower than the FLOPs of some other models, but they are still higher than MDPC-Net’s FLOPs in those datasets. This comparison highlights MDPC-Net’s superior balance of computational efficiency and accuracy, making it particularly well-suited for large-scale remote sensing tasks that demand both high accuracy and low computational overhead. WD-SSMamba, with its lighter parameter count, excels in compactness but at the cost of a slightly higher computational cost in certain configurations, demonstrating a trade-off between model efficiency and accuracy.

## 5 Conclusion

In this study, we proposed MDPC-Net, a hyperspectral image crop classification (HSICC) framework that achieves high classification accuracy while maintaining low computational complexity. By integrating multi-dimensional feature extraction, a linear-projection feature pyramid, and Transformer-based global modeling, MDPC-Net demonstrates strong and stable performance across diverse agricultural scenes.

First, the MDFEM module adopts a three-branch architecture with progressive dilated convolutions to jointly capture spectral, spatial, and spectral–spatial characteristics. This design effectively alleviates long-standing challenges such as “same spectrum, different objects” (e.g., broad- vs. narrow-leaf soybeans) and “same object, different spectra” (e.g., rice vs. rice stubble). Second, the LPFPM module reorganizes and fuses multi-dimensional features through depthwise separable convolutions and linear projection, constructing a feature pyramid that reduces hyperspectral redundancy while enabling multi-scale information fusion. This mechanism supports accurate modeling across spatial scales, from fine-grained plot boundaries (e.g., sesame field edges) to large-scale semantic patterns (e.g., corn distribution). Finally, the Transformer component captures long-range contextual dependencies via self-attention, further enhancing global consistency and boundary delineation in complex agricultural environments.

Importantly, the proposed framework is sensor-agnostic in design and does not rely on sensor-specific handcrafted features, making it readily adaptable to hyperspectral data acquired from different platforms, including airborne, UAV-based, and satellite-borne sensors, with varying spectral resolutions and band configurations. The use of linear projection and multi-dimensional feature coupling allows MDPC-Net to flexibly accommodate differences in spectral dimensionality and spatial resolution, which is critical for real-world deployment across heterogeneous data sources. Moreover, MDPC-Net is not tailored to a specific crop type or region, and its effectiveness across three benchmark datasets with distinct crop compositions and planting patterns suggests strong generalization potential to other agricultural regions. This makes the proposed framework particularly suitable for large-scale agricultural mapping and cross-regional applications, where crop types, field geometries, and management practices vary significantly. Experimental results on three representative benchmark datasets consistently demonstrate that MDPC-Net outperforms all comparison models, validating both its effectiveness and robustness under limited-sample conditions.

In future work, MDPC-Net may be further enhanced through additional lightweight optimization and the integration of spatiotemporal information, such as multi-temporal hyperspectral observations. These extensions would further improve adaptability to different sensors and support dynamic crop monitoring, thereby strengthening the practical value of MDPC-Net for precision agriculture and large-scale agricultural management.

## Data Availability

The original contributions presented in the study are included in the article/supplementary material. Further inquiries can be directed to the corresponding author.

## References

[B1] Aasen H. Honkavaara E. Lucieer A. Zarco-Tejada P. J. (2018). Quantitative remote sensing at ultra-high resolution with UAV spectroscopy: A review of sensor technology, measurement procedures, and data correction workflows. Remote Sens. 10, 1091. doi: 10.3390/rs10071091, PMID: 41725453

[B2] Agilandeeswari L. Prabukumar M. Radhesyam V. Phaneendra K. L. N. B. Farhan A. (2022). Crop classification for agricultural applications in hyperspectral remote sensing images. Appl. Sci. 12, 1670. doi: 10.3390/app12031670, PMID: 41725453

[B3] Al Duhayyim M. Alsolai H. Ben Haj Hassine S. S. Alzahrani J. S. Salama A. Motwakel A. . (2023). Automated deep learning driven crop classification on hyperspectral remote sensing images. Comput. Mater. Contin. 74, 3167–3181. doi: 10.32604/cmc.2023.033054, PMID: 40612875

[B4] Ali I. Mushtaq Z. Arif S. D. Algarni A. F. Soliman N. El-Shafai W. (2023). Hyperspectral images-based crop classification scheme for agricultural remote sensing. Comput. Syst. Sci. Eng. 46, 303–319. doi: 10.32604/csse.2023.034374, PMID: 40612875

[B5] Chandrasekharan S. Gomez K. Al-Hourani A. Kandeepan S. Rasheed T. Goratti L. . (2016). Designing and implementing future aerial communication networks. IEEE Commun. Mag. 54, 26–34. doi: 10.1109/MCOM.2016.7470932, PMID: 41116384

[B6] Chen M. Feng S. Zhao C. Qu B. Su N. Li W. . (2024). Fractional fourier-based frequency-spatial–spectral prototype network for agricultural hyperspectral image open-set classification. IEEE Trans. Geosci. Remote Sens. 62, 1–14. doi: 10.1109/TGRS.2024.3386566, PMID: 41116384

[B7] Fernandes F. C. Faramarzi E. Li X. Ma Z. Ducloux X. (2019). Mobile display power reduction for video using standardized metadata. IEEE Trans. Mob. Comput. 18, 165–178. doi: 10.1109/TMC.2018.2829185, PMID: 41116384

[B8] Gallo I. Ranghetti L. Landro N. La Grassa R. Boschetti M. (2023). In-season and dynamic crop mapping using 3D convolution neural networks and sentinel-2 time series. ISPRS J. Photogramm. Remote Sens. 195, 335–352. doi: 10.1016/j.isprsjprs.2022.12.005, PMID: 41756733

[B9] Gao F. Anderson M. C. Zhang X. Yang Z. Alfieri J. G. Kustas W. P. . (2017). Toward mapping crop progress at field scales through fusion of Landsat and MODIS imagery. Remote Sens. Environ. 188, 9–25. doi: 10.1016/j.rse.2016.11.004, PMID: 41756733

[B10] Guerri M. F. Distante C. Spagnolo P. Bougourzi F. Taleb-Ahmed A. (2024). Deep learning techniques for hyperspectral image analysis in agriculture: A review. ISPRS Open J. Photogramm. Remote Sens. 12, 100062. doi: 10.1016/j.ophoto.2024.100062, PMID: 41756733

[B11] Guo X. Feng Q. Guo F. (2025). CMTNet: a hybrid CNN-transformer network for UAV-based hyperspectral crop classification in precision agriculture. Sci. Rep. 15, 12383. doi: 10.1038/s41598-025-97052-w, PMID: 40216979 PMC11992135

[B12] Hamidi M. Safari A. Homayouni S. (2021). An auto-encoder based classifier for crop mapping from multitemporal multispectral imagery. Int. J. Remote Sens. 42, 986–1016. doi: 10.1080/01431161.2020.1820619, PMID: 41735180

[B13] Howard A. G. Zhu M. Chen B. Kalenichenko D. Wang W. Weyand T. . (2017). MobileNets: efficient convolutional neural networks for mobile vision applications. doi: 10.48550/arXiv.1704.04861, PMID: 41363103

[B14] Hu W. Huang Y. Wei L. Zhang F. Li H. (2015). Deep convolutional neural networks for hyperspectral image classification. J. Sens. 2015, 258619. doi: 10.1155/2015/258619

[B15] Iandola F. N. Han S. Moskewicz M. W. Ashraf K. Dally W. J. Keutzer K. (2016). SqueezeNet: AlexNet-level accuracy with 50x fewer parameters and <0.5MB model size. doi: 10.48550/arXiv.1602.07360, PMID: 41363103

[B16] Khan H. R. Gillani Z. Jamal M. H. Athar A. Chaudhry M. T. Chao H. . (2023). Early identification of crop type for smallholder farming systems using deep learning on time-series sentinel-2 imagery. Sensors 23, 1779. doi: 10.3390/s23041779, PMID: 36850377 PMC9967001

[B17] Khan U. Khan M. Latif M. Naveed M. Alam M. Khan S. . (2024). A systematic literature review of machine learning and deep learning approaches for spectral image classification in agricultural applications using aerial photography. Comput. Mater. Contin. 78, 2967–3000. doi: 10.32604/cmc.2024.045101, PMID: 40612875

[B18] Konduri V. S. Kumar J. Hargrove W. W. Hoffman F. M. Ganguly A. R. (2020). Mapping crops within the growing season across the United States. Remote Sens. Environ. 251, 112048. doi: 10.1016/j.rse.2020.112048, PMID: 41756733

[B19] Liang J. Yang Z. Bi Y. Qu B. Liu M. Xue B. . (2024). A multitree genetic programming-based feature construction approach to crop classification using hyperspectral images. IEEE Trans. Geosci. Remote Sens. 62, 1–17. doi: 10.1109/TGRS.2024.3415773, PMID: 41116384

[B20] Lu Q. Xie Y. Wei L. Wei Z. Tian S. Liu H. . (2024). Extended attribute profiles for precise crop classification in UAV-borne hyperspectral imagery. IEEE Geosci. Remote Sens. Lett. 21, 1–5. doi: 10.1109/LGRS.2023.3348462, PMID: 41116384

[B21] Mei S. Song C. Ma M. Xu F. (2022). Hyperspectral image classification using group-aware hierarchical transformer. IEEE Trans. Geosci. Remote Sens. 60, 1–14. doi: 10.1109/TGRS.2022.3207933, PMID: 41116384

[B22] Michelon G. K. Assunção W. K. G. Grünbacher P. Egyed A. (2023). “ Analysis and propagation of feature revisions in preprocessor-based software product lines,” in 2023 IEEE International Conference on Software Analysis, Evolution and Reengineering (SANER). (Piscataway, NJ, USA: IEEE) 284–295. doi: 10.1109/SANER56733.2023.00035, PMID:

[B23] Nazeri K. Ng E. Joseph T. Qureshi F. Ebrahimi M. (2019). “ EdgeConnect: structure guided image inpainting using edge prediction,” in 2019 IEEE/CVF International Conference on Computer Vision Workshop (ICCVW). (Piscataway, NJ, USA: IEEE) 3265–3274. doi: 10.1109/ICCVW.2019.00408, PMID:

[B24] Roy S. K. Deria A. Shah C. Haut J. M. Du Q. Plaza A. (2023). Spectral–spatial morphological attention transformer for hyperspectral image classification. IEEE Trans. Geosci. Remote Sens. 61, 1–15. doi: 10.1109/TGRS.2023.3242346, PMID: 41116384

[B25] Roy S. K. Krishna G. Dubey S. R. Chaudhuri B. B. (2020). HybridSN: exploring 3-D–2-D CNN feature hierarchy for hyperspectral image classification. IEEE Geosci. Remote Sens. Lett. 17, 277–281. doi: 10.1109/LGRS.2019.2918719, PMID: 41116384

[B26] Sun L. Zhao G. Zheng Y. Wu Z. (2022). Spectral–spatial feature tokenization transformer for hyperspectral image classification. IEEE Trans. Geosci. Remote Sens. 60, 1–14. doi: 10.1109/TGRS.2022.3144158, PMID: 41116384

[B27] Tang H. Yang X. Tang D. Dong Y. Zhang L. Xie W. (2024). Tensor-based few-shot learning for cross-domain hyperspectral image classification. Remote Sens. 16, 4149. doi: 10.3390/rs16224149, PMID: 41725453

[B28] Tu B. Liao X. Li Q. Peng Y. Plaza A. (2022). Local semantic feature aggregation-based transformer for hyperspectral image classification. IEEE Trans. Geosci. Remote Sens. 60, 1–15. doi: 10.1109/TGRS.2022.3201145, PMID: 41116384

[B29] Ullah F. Ullah I. Khan K. Khan S. Amin F. (2025). Advances in deep neural network-based hyperspectral image classification and feature learning with limited samples: a survey. Appl. Intell. 55, 370. doi: 10.1007/s10489-024-06139-w, PMID: 41758449

[B30] Wang S. Liu Z. Chen Y. Hou C. Liu A. Zhang Z. (2023). Expansion spectral–spatial attention network for hyperspectral image classification. IEEE J. Sel. Top. Appl. Earth Obs. Remote Sens. 16, 6411–6427. doi: 10.1109/JSTARS.2023.3288521, PMID: 41116384

[B31] Zhang B. Chen Y. Li Z. Xiong S. Lu X. (2024). SANet: A self-attention network for agricultural hyperspectral image classification. IEEE Trans. Geosci. Remote Sens. 62, 1–15. doi: 10.1109/TGRS.2023.3341473, PMID: 41116384

[B32] Zhang J. Meng Z. Zhao F. Liu H. Chang Z. (2022). Convolution transformer mixer for hyperspectral image classification. IEEE Geosci. Remote Sens. Lett. 19, 1–5. doi: 10.1109/LGRS.2022.3208935, PMID: 41116384

[B33] Zhang H. Xu X. Li S. Plaza A. (2025). Wavelet decomposition-based spectral–spatial mamba network for hyperspectral image classification. IEEE Trans. Geosci. Remote Sens. 63, 1–17. doi: 10.1109/TGRS.2025.3590154, PMID: 41116384

[B34] Zhao Z. Xu X. Li S. Plaza A. (2024). Hyperspectral image classification using groupwise separable convolutional vision transformer network. IEEE Trans. Geosci. Remote Sens. 62, 1–17. doi: 10.1109/TGRS.2024.3377610, PMID: 41116384

[B35] Zunair H. Rahman A. Mohammed N. Cohen J. P. (2020). Uniformizing techniques to process CT scans with 3D CNNs for tuberculosis prediction. doi: 10.48550/arXiv.2007.13224, PMID: 41363103

